# Treatment with Volanesorsen, a 2′-O-Methoxyethyl-Modified Antisense Oligonucleotide Targeting *APOC3* mRNA, Does Not Affect the QTc Interval in Healthy Volunteers

**DOI:** 10.1089/nat.2019.0837

**Published:** 2020-08-06

**Authors:** Lynnetta M. Watts, Ewa Karwatowska-Prokopczuk, Eunju Hurh, Veronica J. Alexander, Kristin Balogh, Louis O'Dea, Richard S. Geary, Sotirios Tsimikas

**Affiliations:** ^1^ Clinical Development, Ionis Pharmaceutical, Inc, Carlsbad, California, USA.; ^2^ Clinical Development, Akcea Therapeutic, Boston, Massachusetts, USA.; ^3^ Division of Cardiovascular Medicine, University of California San Diego, La Jolla, California, USA.

**Keywords:** QTc interval, antisense oligonucleotide, subcutaneous (SC) therapeutic dose, supratherapeutic dose, volanesorsen

## Abstract

The aim of this study was to assess the effect of volanesorsen on the corrected QT (QTc) interval. This thorough QT study enrolled 52 healthy male and female subjects who were randomized at a single site in a four-way crossover study. Subjects were randomly assigned to 1 of 12 treatment sequences and crossed over into four treatment periods over the course of which each subject was to receive a single therapeutic dose of volanesorsen as a 300 mg subcutaneous (SC) injection, a single supratherapeutic dose of volanesorsen as 300 mg intravenous (IV) infusion, a single oral (PO) dose of moxifloxacin (positive control), and placebo dose. The study demonstrated that volanesorsen 300 mg SC and 300 mg IV did not have a clinically relevant effect on ΔΔQTcF exceeding 10 ms. The largest mean effect at any postdose time point was 3.0 ms (90% confidence interval [CI]: 0.8–5.2) after SC dosing and 1.8 ms (90% CI −0.4 to 4.0) after IV dosing. Volanesorsen, at the studied therapeutic and supratherapeutic doses, does not have a clinically meaningful effect on the QTc.

## Introduction

Volanesorsen is a second-generation antisense oligonucleotide (ASO) drug targeted to human *APOC3* mRNA. The hybridization of volanesorsen to the cognate mRNA results in RNase H1-mediated degradation of the *APOC3* mRNA, thus preventing production of the apoC-III protein. Maximal antisense-mediated reduction of target mRNA levels is typically greater than 90% of control levels in sensitive tissues [1–3]. Furthermore, reduction in target mRNA levels using this approach correlates directly with a subsequent reduction in target protein levels.

APOC-III is a major regulator of lipoprotein metabolism and plays a pivotal role in regulating plasma triglyceride levels [4,5]. It is a component of triglyceride-rich lipoproteins (TRLs), a potent inhibitor of lipoprotein lipase (LPL), and delays clearance of TRLs, resulting in hypertriglyceridemia [6].

Volanesorsen is being developed for reduction of triglyceride levels in patients with familial chylomicronemia syndrome (FCS), a rare autosomal recessive disorder characterized by severe hypertriglyceridemia and recurrent pancreatitis due to a deficiency in LPL or associated proteins [7]. All clinical trials of volanesorsen have shown potent and clinically meaningful reductions in fasting plasma apoC-III and triglyceride levels (∼80% and 70%, respectively, mean percent reduction from baseline with 300 mg dose) with a very high degree of consistency of response between the different patient groups [8]. In May 2019, volanesorsen (Waylivra™) was approved for clinical use in patients with FCS by the European Medicines Agency [9].

Preclinically, the human ether-a-go-go potassium channel (hERG channel) and animal telemetry studies have demonstrated that second-generation ASOs do not interfere with hERG channel proteins or cause QT prolongation in animal models [10]. Details of hERG channel study can be found in Supplementary Data S1 and details for animal telemetry studies for volanesorsen can be found in Supplementary Data S2 and Supplementary Table S1. Until recently, health authorities required that ASOs and small-molecule studies should be performed to determine whether corrected QT (QTc) prolongation occurs in humans. Waivers for Spinraza™ and Tegsedi™ [11] were recently granted by the health authority agencies during phase III or before marketing authorization submission. At the time this QTc study was initiated, no specific regulations were available for ASOs, and therefore, the study was conducted to assess the QTc interval effect of volanesorsen administered as a 300 mg subcutaneous (SC) therapeutic and a 300 mg intravenous (IV; 2-h infusion) supratherapeutic dose relative to placebo in healthy adult male and female subjects.

## Materials and Methods

### ASO volanesorsen

Volanesorsen is a synthetic oligomer of 20 nucleotides (ie, a 20-mer) that are connected sequentially by phosphorothioate linkages. The nucleotide sequence of volanesorsen is AGCTT CTTGTCCAGC TTTAT and is complementary to a 20-nucleotide stretch within the 3′ untranslated region of the *APOC3* mRNA transcript at base position 489–508. Structurally, the oligonucleotide has three regions: the five nucleotides at the 5′ end and the five nucleotides at the 3′ end are composed of 2′-*O*-(2-methoxyethyl) (MOE)-modified ribonucleotides. These MOE-modified nucleotides confer (1) increased affinity to the target mRNA [12,13]; (2) increased resistance to exonucleases and endonucleases (thereby increasing stability in tissue) [14], and (3) amelioration of some of the high-dose toxicities, such as activated partial thromboplastin time prolongation and complement activation [15,16], thereby resulting in an improved safety profile compared with first-generation antisense drugs containing phosphorothioate-modified oligodeoxynucleotides (DNA) [17,18]. The central portion of the oligonucleotide (underlined above) is composed of 10 oligodeoxynucleotides. This chimeric design is called an MOE-Gapmer, and volanesorsen uses this chimeric structure to enable use of the RNase H1-mechanism for antisense activity. Volanesorsen was formulated in 0.9% sodium chloride (200 mg/mL) and was provided by IONIS Pharmaceuticals, Inc.

### Study design

This was a Phase 1, randomized, double-blind, placebo-controlled, single-site, four-way crossover study in healthy male and female subjects designed to determine if volanesorsen administered as a single therapeutic dose (300 mg SC) and a single supratherapeutic dose (300 mg IV) delayed cardiac repolarization as determined by the measurement of QT/QTc interval. Further details of the study design and treatment sequences can be found in Supplementary Data S3 and Supplementary Table S2.

Approximately 65 healthy male and female subjects aged 18 to 55 years, inclusive, were randomized into the study at a single site in this four-way crossover study, of which 52 subjects were evaluable and completed treatment. This study was performed according to the amended Declaration of Helsinki and the appropriate institutional review board approved the protocol. Informed consent was obtained from all individual participants included in the study. Subjects were randomly assigned to 1 of 12 treatment sequences and crossed over into four treatment periods over the course of which each subject was to receive a single SC injection of volanesorsen, a single IV infusion of volanesorsen, a single oral (PO) dose of moxifloxacin, and placebo administered as the following combinations according to the randomization schedule: (1) 300 mg volanesorsen IV/placebo SC, (2) 300 mg volanesorsen SC/placebo IV, (3) 400 mg moxifloxacin PO/placebo IV+placebo SC, and (4) placebo IV/placebo SC. The study consisted of four treatment periods. The first treatment period consisted of four phases: a screening phase (days −32 to −2), an admission phase (day −1), a treatment phase (day 1), and a follow-up phase (day 2). Periods 2, 3, and 4 each consisted of three phases: an admission phase (day −1), a treatment phase (day 1), and a follow-up phase (day 2).

On day 1 of each treatment period, subjects were connected to a continuous 12-lead ECG Holter monitoring (Mortara™ surveyor systems) device for 25 h starting ∼1 h before dose initiation. Up to 10 replicate ECGs were extracted at three time points before dosing (−45, −30, and −15 min) and 0.5, 1, 1.5, 2 (immediately before the end of volanesorsen or placebo infusion), 2.5, 3, 3.5, 4, 5, 6, 8, 10, 12, and 24 h after the start of the infusion, for a total of 17 time points. The ECG extractions were time matched to the pharmacokinetics (PK) samples but were obtained before the actual plasma sampling time. The study used a standard crossover thorough QT (TQT) design as per the International Conference on Harmonization (ICH) E14 guideline entitled, “Guidance for Industry: The Clinical Evaluation of QT/QTc Interval Prolongation and Proarrhythmic Potential for Non-Antiarrhythmic Drugs” [19] using a 7-day washout to prevent carryover effects. Subjects were randomized to treatment sequences in an effort to ensure that baseline characteristics were evenly distributed across study groups. Volanesorsen and placebo treatments were administered under a double-blind to control for bias in cardiodynamic and safety assessments, while moxifloxacin was administered in an open-label manner for sensitivity analysis. Because it was not feasible to blind the investigator and subjects to the route of administration, a placebo SC injection or placebo IV infusion was administered with each active volanesorsen dose. Again, to protect the blind, the placebo treatment involved administration of placebo via both SC and IV routes. Moxifloxacin was coadministered with a placebo SC injection and IV infusion to maintain consistent treatment procedures across study periods. The volanesorsen 300 mg SC injection served as the therapeutic dose, while the 300 mg IV infusion served as the supratherapeutic dose. QT interval corrected using Fridericia's formula (QTcF) was calculated, and change-from-baseline QTcF (ΔQTcF) analysis was calculated using baseline QTcF defined based on three predose time points in each period. The single-dose design allowed for ECG measurements to be extracted at time points in the range of observed therapeutic plasma concentrations and limited unnecessary exposure to study drug.

### Subject population analyzed

1. Safety population: All subjects who were randomized and received at least one dose of study drug.
2. PK population: All subjects who were randomized and received at least one dose of volanesorsen and had at least one evaluable concentration result.
3. QT/QTc population: All subjects who are in the safety population with measurements at baseline as well as on-treatment with at least one postdose time point with a valid ΔQTcF value.
4. PK/QTc population: All subjects who were in both the QT/QTc and PK populations with at least one pair of postdose PK and QTcF data from the same time point.

### Volanesorsen concentration in plasma

Subjects had serial blood samples collected for PK analysis on day 1 of each period before initiation of dosing through 24 h after initiation of dosing.

Plasma and urine samples were analyzed by PPD (Richmond, VA) using a validated hybridization enzyme-linked immunosorbent assay method. The quantification range of the assay was 1.00 to 100 ng/mL, with the low and high ends of this range defining the lower limit of quantification and the upper limit of quantification [20].

### Safety and tolerability

The safety and tolerability of each subject were determined by (1) recording of nonserious adverse events, (2) vital signs including body temperature, respiratory rate, blood pressure, and pulse, (3) clinical laboratory evaluations that began after the subject signed the informed consent form and stopped at the end of the subject's follow-up period.

### Pharmacokinetic analysis

The pharmacokinetic parameters of volanesorsen were calculated by noncompartmental analysis using Phoenix WinNonlin Version 7.0 (Pharsight Corporation, Mountain View, CA). The maximum observed drug concentration in plasma (*C*_max_) and the time to reach *C*_max_ (*T*_max_) were obtained directly from the observed concentration/time data. Area under the plasma concentration/time curve from time 0 up to 24 h (AUC_0–24h_) was calculated using the linear trapezoidal rule. The absolute bioavailability after SC administration, as an approximation, was calculated as the ratio of AUC_0–24h_ after SC and AUC_0–24h_ after IV.

### Pharmacodynamic statistical methodology

All analyses were performed by a core ECG laboratory (iCardiac Technologies, Inc.) with expertise in analysis and interpretation of cardiac electrophysiology data.

### By time point analysis

The primary analysis for QTcF was based on Model 1 with ΔQTcF as the dependent variable; period, sequence, time (categorical), treatment (therapeutic and supratherapeutic doses of volanesorsen, moxifloxacin, and placebo), and time-by-treatment interaction as fixed effects; and baseline QTcF as covariate. Baseline was the average of the three predose time points (−45, −30, and −15 min) on day 1 in each period. Subject was included as a random effect for the intercept. An unstructured covariance matrix was specified for the repeated measures at postdose time points for subjects within the treatment period. If the model with unstructured covariance matrix failed to converge, other covariance matrices such as autoregressive and compound symmetry were considered. From this analysis, the least-squares (LS) mean and two-sided 90% confidence intervals (CIs) were calculated for the contrast “volanesorsen vs. placebo” at each dose of volanesorsen and each postdose time point, separately. If the upper bound of the two-sided 90% CI fell below 10 ms at all postdose time points, it was concluded that volanesorsen did not prolong the QTc interval to a clinically meaningful degree, that is, a negative TQT study.

### Assay sensitivity

The analysis to show assay sensitivity was based on the change-from-baseline values after dosing moxifloxacin. The same model was used as described for the primary analysis. For the time points 2, 3, and 4 h, the contrast in treatment placebo-adjusted change-from-baseline QTcF (ΔΔQTcF) = “moxifloxacin – placebo” was tested against the one-sided null hypothesis ΔΔQTcF ≤5 ms at a 5% significant level. Multiplicity was controlled by using a Hochberg procedure. If after this procedure ΔΔQTcF was significantly larger than 5 ms for at least one time point, assay sensitivity was considered to be shown. In addition, two-sided 90% CIs were obtained for the contrast at all time points for descriptive purposes and used in the figures.

### Secondary analysis

For HR, PR, and QRS intervals, the analysis was based on the change-from-baseline postdosing (ΔHR, ΔPR, ΔQRS). The same model was used as described for QTcF. Plots of each of the above parameters are located in Supplementary Figure S1.

#### Exposure/response analysis

The relationship between volanesorsen plasma concentration and ΔΔQTcF was investigated by linear mixed-effects modeling. Three linear models were considered:

1. Model 1 was a linear model with an intercept
2. Model 2 was a linear model with mean intercept fixed to 0 (with variability)
3. Model 3 was a linear model with no intercept.

Time-matched concentration was included in the model as a covariate and subject as a random effect for intercept and slope, where applicable. The model that fits the data best—that is, had the smallest Akaike information criterion (AIC) and the model predicted CIs similar to the observed CIs [21], was used for predicting population average ΔΔQTcF and its corresponding 90% two-sided CI at the geometric mean peak volanesorsen concentration at the 300 mg SC therapeutic dose and 300 mg IV supratherapeutic dose. The analyses were performed using the PK/QTc population.

Supplementary Table S3 summarizes the result of the volanesorsen concentration ΔΔQTcF analysis. Model 1 was used for further analysis since the model with an intercept was found to fit the data best based on the AIC values among the three candidate models: 6420, 6419, and 6540, respectively. Among them, the AIC value for Model 1 was somewhat larger than that for Model 2 (which had the smallest AIC among the three candidate models). Model 1 was chosen as the final model by considering the *P*-values for the slopes and the model forms. The plots of the standardized residuals versus the fitted ΔΔQTcF values and volanesorsen concentrations showed that the standardized residuals are distributed symmetrically around zero, which indicates the appropriateness of Model 1 and does not indicate any significant departure from model assumptions for within-event errors. Similarly, the normal QQ plots of the standardized residuals and the random effects did not show any violations of the normality assumption for the within-subject errors and the random effects. Thus, Model 1 was considered appropriate to describe the concentration/ΔΔQTcF relationship.

A time delay between plasma concentration of volanesorsen and ΔΔQTcF was not observed, and therefore, time-matched concentration was included in the model as a covariate and subject as a random effect for intercept and slope, where applicable.

The plot of the observed median-quantile volanesorsen concentrations and associated mean ΔΔQTcF (90% CI) together with the mean (90% CI) predicted ΔΔQTcF were used to evaluate the adequacy of the model fit to the assumption of linearity and the impact on quantifying the concentration/response relationship.

Model 1 was used for predicting population average ΔΔQTcF and its corresponding 90% two-sided CI at the geometric mean peak volanesorsen concentration at the 300 mg SC therapeutic dose and 300 mg IV supratherapeutic dose.

## Results

### Safety and tolerability

Single 300 mg doses of volanesorsen were generally safe and well-tolerated in healthy subjects when administered as the therapeutic SC and supratherapeutic IV dose. Overall, 27 (58%) subjects experienced a total of 64 adverse events (AEs) following administration of 300 mg volanesorsen SC, 9 (19%) subjects experienced a total of 15 AEs following administration of 300 mg volanesorsen IV, 7 (15%) subjects experienced a total of 11 AEs following administration of placebo, and 8 (16%) subjects experienced 14 AEs following administration of 400 mg moxifloxacin PO. All AEs reported following administration of 300 mg volanesorsen were mild in severity and the majority were considered to be potentially related to study drug (26 subjects with 61 AEs following SC administration and 7 subjects with 12 AEs following IV administration). One subject experienced two severe AEs following placebo (elevated creatinine phosphokinase and aspartate aminotransferase due to strenuous physical activity). The most frequently experienced AEs, regardless of causality, as well as those considered to be potentially related to study drug, were general disorders and administration-site conditions, reported for 56% and 15% of subjects, respectively, following volanesorsen 300 mg SC and IV.

### Pharmacokinetic evaluation

**FIG. 1.**
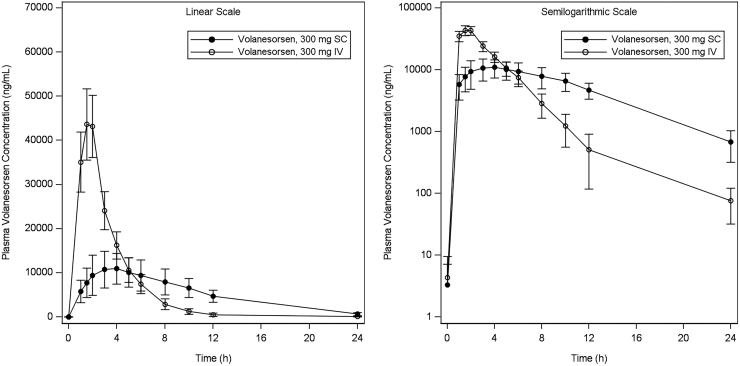
Mean (±SD) plasma concentration of volanesorsen versus time following single SC and IV doses. IV, intravenous; SC, subcutaneous; SD, standard deviation.

The arithmetic mean ± SD concentration/time data for volanesorsen are shown in Fig. 1. Volanesorsen was rapidly absorbed into the systemic circulation after SC administration, with the median peak plasma level observed 4 h after dosing. Following a 2-h IV infusion, the peak plasma level was attained at the end of the infusion. After reaching peak concentrations, mean plasma concentrations declined rapidly.

Summary data for plasma PK parameters by treatment are provided in Table 1. The geometric mean AUC_0–24h_ values for volanesorsen were 123 and 155 μg·h/mL after a single therapeutic dose (300 mg SC) and supratherapeutic dose (300 mg IV) of volanesorsen, respectively. The geometric mean absolute bioavailability (*F*) for the SC dose of 300 mg based on AUC_0–24h_ was 78.7%, which is likely a conservative estimate as AUC_0–24h_ is a partial AUC and the plasma volanesorsen concentration at 24 h postdose for the SC dose was higher than that for the IV dose. The geometric mean *C*_max_ values were 11.1 and 45.0 μg/mL for the 300 mg SC and 300 mg IV doses, respectively. The *C*_max_ was ∼4.1-fold higher after IV infusion compared with SC injection of volanesorsen based on the comparison of geometric means. Higher variability was associated with *C*_max_ and AUC after SC dosing (geometric %CV = 38.5 and 30, respectively) compared with that after IV dosing (geometric %CV = 17.4% and 16.6%, respectively).

**Table 1.** Summary of Plasma Pharmacokinetic Parameters of Volanesorsen

Treatment	Statistic	C_max_ (μg/mL)	T_max_ (h)	AUC_0–24h_ (μg·h/mL)	F (%)
Volanesorsen 300 mg SC	*n*	46	46	46	45^a^
	Geometric mean	11.1	4.08^b^	123	78.7
	Geometric %CV	38.5	1.58–10.1^c^	30.5	21.3
Volanesorsen 300 mg IV	*n*	48	48	48	NA
	Geometric mean	45.0	2.08^b^	155	NA
	Geometric %CV	17.4	1.58–2.17^c^	16.6	NA

^a^ The *F* was not available for one subject who did not receive an IV dose of volanesorsen.

^b^ Median is shown for *T*_max_.

^c^ Min–max is shown for *T*_max_.

AUC_0–24h_, area under the plasma concentration/time curve from time zero to 24 h; *C*_max_, maximum observed concentration; *F*, absolute bioavailability after SC administration calculated as AUC_0–24h_ after SC administration divided by AUC_0–24h_ after 2-h IV infusion; IV, intravenous; NA, not applicable; SC, subcutaneous; *T*_max_, time of *C*_max_.

### By time point analysis

**FIG. 2.**
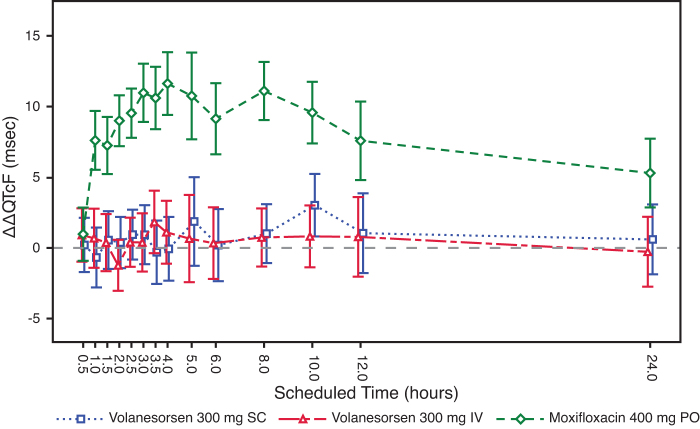
Least squares mean (±90% CI) placebo-corrected change from baseline QTcF across time points. CI, confidence interval.

Following administration of a single 400 mg PO dose of moxifloxacin, as a positive control, the lower bound of the two-sided 90% CI for the LS mean difference in ΔQTcF between moxifloxacin and placebo exceeded 5 ms beginning at the 1-h postdose time point through the 10-h postdose time point, suggesting that sufficient assay sensitivity was achieved. Volanesorsen 300 mg SC and IV administration did not have an effect on the HR, supporting use of QTcF as the primary endpoint. The ΔΔQTcF data over 24-h time points are shown in Fig. 2.

Following SC or IV administration of volanesorsen, the upper bound of the two-sided 90% CI for the LS mean difference in changes from baseline in QTcF between volanesorsen and placebo was less than 10 ms at all time points assessed. Therefore, this study met the criteria described by the ICH E14 guidance as a “negative thorough QT/QTc study” (ICH E14 2005).

The ΔΔQTcF varied with small mean changes in a way that does not suggest that volanesorsen has any effect on this interval. The largest mean ΔΔQTcF was only 3.0 ms (90% CI 0.8–5.2) 10 h after dosing with volanesorsen 300 mg SC, and 1.8 ms (90% CI −0.4 to 4.0) 3.5 h after dosing with 300 mg IV. Occasional biphasic or notched T-waves were observed both for placebo and active treatment.

Volanesorsen 300 mg SC and IV did not have a clinically meaningful effect on the PR or QRS intervals.

All mean placebo-corrected ΔPR values on both volanesorsen doses were within −3.2 to 1.1 ms. Volanesorsen at the studied doses did not have an effect on the QRS interval with mean ΔΔQRS within ±1.0 ms at all postdose time points. There were no categorical outliers for PR or QRS intervals (data not shown).

#### Concentration/effect analysis

**FIG. 3.**
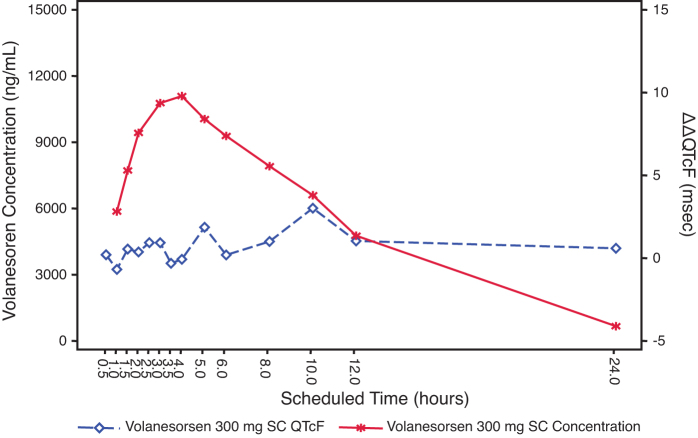
Joint plot of mean volanesorsen 300 mg SC plasma concentration and mean ΔΔQTcF over time (PK/QTc population).

**FIG. 4.**
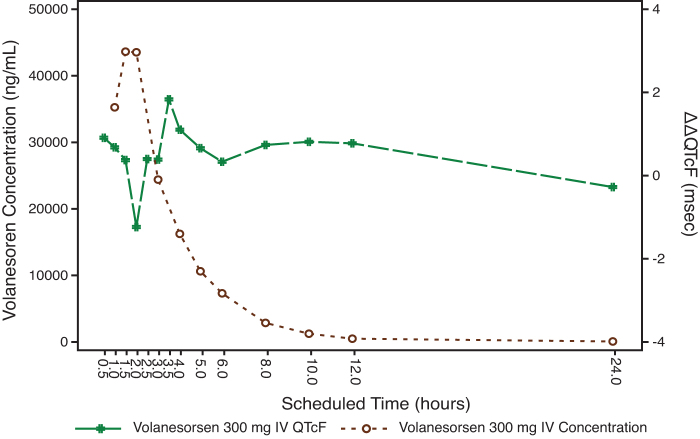
Joint plot of mean volanesorsen 300 mg IV plasma concentration and mean ΔΔQTcF over time (PK/QTc population).

**FIG. 5.**
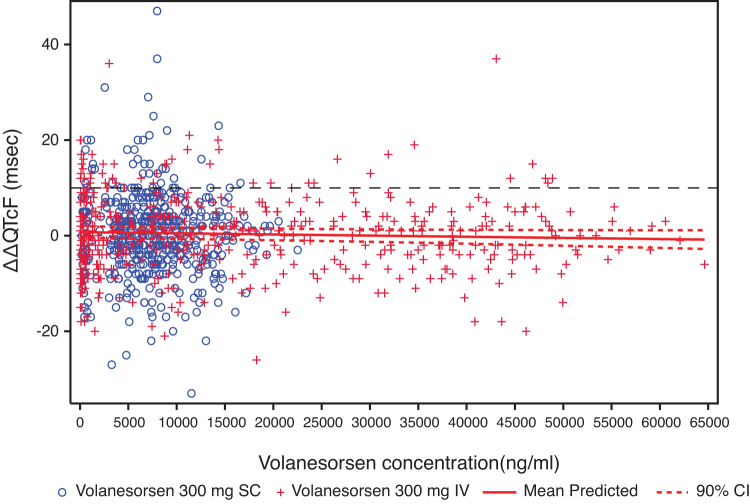
Relationship between individual volanesorsen concentrations and ΔΔQTcF with 90% CI (PK/QTc population). Prediction was based on Model 1.

**FIG. 6.**
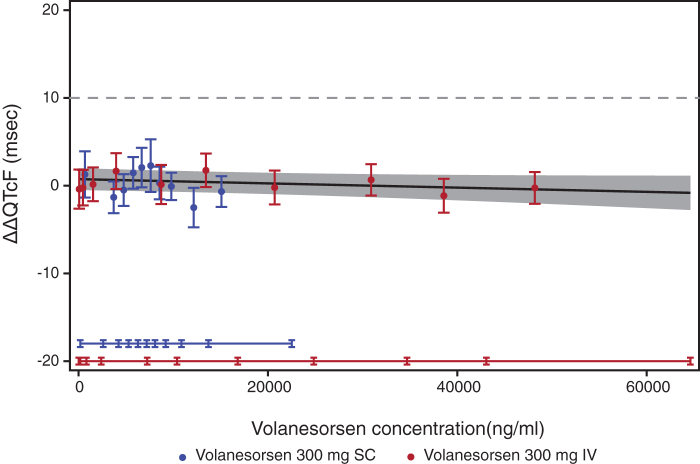
Goodness-of-fit plot for observed and predicted relationship between volanesorsen levels and ΔΔQTcF (PK/QTc population). Analysis was based on PK/QTc population. *Blue* and *red circles* with *vertical bars* denote the observed mean ΔΔQTcF with 90% CI displayed at the median plasma concentration within each decile of volanesorsen on doses of 300 mg SC and 300 mg IV, respectively. The *solid black line* with *gray shaded area* denotes the model-predicted mean ΔΔQTcF with 90% CI. The *horizontal blue* and *red lines* with notches show the range of plasma concentrations divided into deciles for volanesorsen on doses of 300 mg SC and 300 mg IV, respectively.

The mean plasma concentration/time profiles of volanesorsen and mean ΔΔQTcF over the scheduled time after 300 mg SC and 300 mg IV are shown in Figs. 3 and 4. The relationship between the individually observed volanesorsen plasma concentrations and ΔΔQTcF along with Model 1 and 90% CI is visualized in Fig. 5. A final assessment of the adequacy of the Model 1 is given by the goodness-of-fit plot in Fig. 6, which shows the mean ΔΔQTcF (90% CI) within each volanesorsen plasma concentration decile and the model-predicted mean ΔΔQTcF with 90% CI. The plot shows that the predicted ΔΔQTcF values are close to the observed values. Therefore, it can be concluded that the linear mixed-effects model adequately describes the relationship between ΔΔQTcF and volanesorsen concentrations. The estimated slope of the exposure/response relationship was −0.000024 ms per ng/mL (90% CI −0.000054 to 0.000006) with an intercept of 0.742 ms. The slope of the relationship was not statistically significant and a concentration-dependent effect of volanesorsen on QTcF was therefore not identified.

The predicted ΔΔQTcF at the geometric mean peak volanesorsen plasma concentration is shown in Table 2. The predicted ΔΔQTcF based on the model was 0.48 ms (90% CI −0.74 to 1.69) and −0.34 ms (90% CI −1.89 to 1.21) at the observed geometric mean *C*_max_ after volanesorsen 300 mg SC and 300 mg IV (4.1-fold of geometric mean *C*_max_ for 300 mg SC), respectively.

**Table 2.** Predicted ΔΔQTcF Interval at Geometric Mean Peak Volanesorsen Concentration from Model 1 (PK/QTc Population)

Treatment	Geometric mean (90% CI^a^) of* C*_max_ (ng/mL)	Predicted ΔΔQTcF (ms)	90% CI of predicted ΔΔQTcF (ms)
Volanesorsen 300 mg SC	11,013 (10,023 to 12,102)	0.48	−0.74 to 1.69
Volanesorsen 300 mg IV	44,866 (42,880 to 46,943)	−0.34	−1.89 to 1.21

^a^ The 90% CI of the geometric mean was calculated in the logarithmic domain and presented after back-transformation to the original concentration domain.

## Discussion

The primary objective of this study was to assess the QTc effect of volanesorsen administered as a 300 mg SC therapeutic and a 300 mg IV supratherapeutic dose relative to placebo in healthy adult male and female subjects. This study also evaluated the effect of volanesorsen on other ECG parameters (HR, PR, and QRS interval) and the assay sensitivity to detect a change in the QTc interval, using 400 mg moxifloxacin as the active control. In addition, the PK of volanesorsen when administered as a single therapeutic 300 mg SC and a single supratherapeutic 300 mg IV dose was examined. Finally, this study assessed the safety of volanesorsen when administered as a single therapeutic 300 mg SC and a single supratherapeutic 300 mg IV dose.

This TQT study demonstrated that volanesorsen 300 mg SC and 300 mg IV did not have a clinically relevant effect on ECG parameters. The pattern of HR change was comparable between volanesorsen and placebo. An effect on ΔΔQTcF exceeding 10 ms can clearly be excluded as the largest mean effect at any postdose time point, 3.0 ms (90% CI 0.8–5.2) after SC dosing and 1.8 ms (90% CI −0.4 to 4.0) after IV dosing. The QT effect of 400 mg moxifloxacin confirmed the study's assay sensitivity with mean ΔΔQTcF at the predefined time points (2, 3, and 4 h) of 9.0, 11.0, and 11.6 ms, respectively, with all lower bounds of the 90% CI above 5 ms.

The slope of the concentration-QTc relationship was shallow and not statistically significant (−0.000024 ms per ng/mL [90% CI −0.000054 to 0.000006]). The predicted ΔΔQTcF effect using the proposed linear model with intercept was 0.48 ms (90% CI −0.74 to 1.69) and −0.34 ms (90% CI −1.89 to 1.21) at the observed geometric mean peak plasma level after dosing with volanesorsen 300 mg SC and 300 mg IV, respectively. The predicted QTc effect was negligible throughout the observed range of plasma concentrations from therapeutic and supratherapeutic doses and the results were consistent with the “by-time point” analysis. The exposure/response analysis provided further evidence to support the conclusion that volanesorsen did not have a clinically meaningful effect on cardiac repolarization.

These data indicate that therapeutic doses of volanesorsen are not expected to cause clinically relevant QTc prolongation, given the fact that even the supratherapeutic dose of 300 mg IV, providing 4.1-fold higher plasma exposure compared with the recommended 300 mg SC dose, did not show an effect on ΔΔQTcF exceeding 10 ms.

The absence of a QTc prolongation effect of volanesorsen is consistent with previously reported results of the TQT study for mipomersen [22] and the exposure/response analysis of a group of 2′-O-MOE ASOs [23]. These cumulative results confirm that 2′-MOE ASOs do not cause QT prolongation in humans at clinically relevant doses, as predicted from both the *in vitro* hERG assay and monkey safety pharmacology studies with telemetry ECG monitoring at high toxicology dose levels. Oligonucleotide drugs are both large and highly charged that limit their potential for direct inhibition of hERG potassium channels, which has been evaluated and verified in both *in vitro* and nonhuman primate studies [24,25]. Considering the low likelihood of QT prolongation for ASOs, further TQT studies may not be warranted for this class of compounds coming to development. Instead, incorporation of ECG monitoring in Phase I studies with matching PK data for exposure/response analysis could be considered as recommended by the new FDA guidance published in 2015 [26,27].

## Conclusion

Volanesorsen, at the studied therapeutic and supratherapeutic doses, did not have a clinically meaningful effect on the QTc interval for single IV or SC doses of 300 mg volanesorsen. The upper bound of the two-sided 90% CI for ΔΔQTcF was less than 10 ms at all time points assessed. Assay sensitivity was adequate to detect a change in the QTc interval as assessed using 400 mg moxifloxacin as the active control. Single IV or SC doses of 300 mg volanesorsen did not affect other ECG parameters, including HR, PR, and QRS, and were generally safe and well-tolerated in healthy subjects.

## Supplementary Material

Supplemental data

Supplemental data

Supplemental data

Supplemental data

Supplemental data
