# Supplementary material for: Treatment with Volanesorsen, a 2′-O-Methoxyethyl-Modified Antisense Oligonucleotide Targeting APOC3 mRNA, Does Not Affect the QTc Interval in Healthy Volunteers
Source: Nucleic Acid Ther. 2020 Aug 6;30(4):198–206. doi: 10.1089/nat.2019.0837 (PMC7415887; doi:10.1089/nat.2019.0837)
Supplement: Supplemental data [file Supp_Data.pdf]

Supplementary Data

Supplementary Data S1. Human HEK293 Assay

Studies demonstrating the *in vitro* effects of volanesorsen on ionic currents in voltage-clamped human embryonic kidney cells (HEK293) that stably express the human ether-a-go-go-related gene (hERG) have been conducted. Formulations were analyzed for stability (nominal 100 and 300 μM), homogeneity (nominal 300 μM), and concentration (nominal 100 and 300 μM). Assayed concentrations from outflow samples were used for the evaluation of dose/response relationship. Volanesorsen inhibited hERG current by (mean ± SEM) −0.6% ± 0.6% at 89.5 μM ($n = 3$) and 0.3% ± 1.0% at 251.0 μM ($n = 3$) versus 0.8% ± 1.4% ($n = 3$) in control. hERG inhibition at 89.5 and 251.0 μM was not statistically significant ($P < 0.05$) when compared with vehicle control values. The IC_{50} for the inhibitory effect of volanesorsen on hERG potassium current could not be calculated but was estimated to be greater than 251.0 μM. Under similar conditions, the positive control (60 nM terfenadine) inhibited hERG potassium current by (mean ± SEM; $n = 2$) 76.2% ± 3.5%, confirming the sensitivity of the test system to hERG inhibition.

Supplementary Data S2. Monkey Telemetry Data

The acute cardiovascular effects of a 1-h intravenous (IV) infusion (12 mg/kg) and a subcutaneous (SC) administration (40 mg/kg) of ISIS 304801 in conscious male telemetered cynomolgus monkeys were evaluated. The study followed a single-dose administration design. On day 1, groups 1 and 2 ($n = 4$/group) received control article/vehicle, sterile phosphate-buffered saline (PBS), by IV infusion (1 h ± 5 min), or ISIS placebo (a PBS solution) by SC injection. On day 3, group 1 animals received volanesorsen (12 mg/kg) administration IV and group 2 animals received volanesorsen (40 mg/kg administered SC). Lead-II ECG parameters [QRS duration, RR, PR, QT (uncorrected) and QTc (rate-corrected) intervals with Bazett (QTcB) and Fridericia (QTcF)] were continuously record/derived. The analyzed time points were 1 h before administration and 2, 4, and 24 h (±30 min) post-dose. There were no significant changes observed in qualitative (Lead-II configuration) ECG assessments, quantitative ECG parameters (QRS duration PR, RR, QT, QTcB, and QTcF intervals between placebo IV or SC treatment and volanesorsen treatment [IV infusion (12 mg/kg), or SC (40 mg/kg)]. Slight changes observed in RR and QT intervals, following treatment, were within the normal range of variation for the species Slight changes in QT intervals were secondary to fluctuations in RR intervals and were minimized following correction of QT for RR (QTcB and QTcF) (Supplementary Table S1).

Supplementary Data S3. Study Design Volanesorsen QTc Study

Subjects were randomized to 1 of 12 treatment sequences by an unblinded pharmacist after all screening assessments had been completed and after the investigator had verified that subjects were eligible for the study. No subject began treatment before randomization and assignment of a unique subject identification number. The 12 treatment sequences are shown in Supplementary Table S2.
