# Supplementary material for: Treatment with Volanesorsen, a 2′-O-Methoxyethyl-Modified Antisense Oligonucleotide Targeting APOC3 mRNA, Does Not Affect the QTc Interval in Healthy Volunteers
Source: Nucleic Acid Ther. 2020 Aug 6;30(4):198–206. doi: 10.1089/nat.2019.0837 (PMC7415887; doi:10.1089/nat.2019.0837)
Supplement: Supplemental data [file Supp_TableS1.pdf]

Supplementary Table S1. QRS, PR, RR, QT, QTcB, and QTcF Mean Data After 1-H Intravenous Infusion at 12 mg/kg and 40 mg/kg SC Injection of Volanesorsen in Conscious Male Telemetered Cynomolgus Monkeys

	Group 1: sterile PBS (IV)		*Group 1: volanesorsen (12 mg/kg, IV)*		*Group 2: placebo solution (SC)*		*Group 2: volanesorsen (40 mg/kg, SC)*	
Time point (h)	*Mean*	*SD*	*Mean*	*SD*	*Mean*	*SD*	*Mean*	*SD*
QRS duration (ms)[a]								
−1	32	3	32	4	37	5	37	6
2	33	5	32	2	37	6	37	6
4	32	5	31	5	36	5	36	7
24	31	5	32	3	36	5	37	6
PR interval (ms)[a]								
−1	87	7	86	8	77	8	75	9
2	85	8	81	8	78	9	73	10
4	86	9	86	10	78	8	77	10
24	84	7	86	10	74	11	74	9
RR interval (ms)[a]								
−1	572	39	569	31	467	73	503	126
2	514	41	501	78	513	59	456	87
4	577	93	540	86	530	75	496	98
24	523	35	574	70	524	143	489	95
QT interval (ms)[a]								
−1	279	39	279	43	224	36	230	47
2	263	20	248	24	223	29	214	24
4	277	24	265	32	230	34	216	32
24	259	31	278	35	235	49	223	39
QTcB (ms)[a]								
−1	370	52	369	49	327	30	324	27
2	368	39	353	36	312	26	318	16
4	367	36	363	45	316	26	307	26
24	360	40	368	43	325	26	319	27
QTcF (ms)[a]								
−1	336	47	336	47	288	33	288	36
2	329	32	314	29	279	27	278	17
4	334	29	326	39	284	30	273	28
24	323	36	335	39	291	35	283	32

[a]$n = 4$.

IV, intravenous; PBS, phosphate-buffered saline; SC, subcutaneous; SD, standard deviation.
