# Supplementary material for: Treatment with Volanesorsen, a 2′-O-Methoxyethyl-Modified Antisense Oligonucleotide Targeting APOC3 mRNA, Does Not Affect the QTc Interval in Healthy Volunteers
Source: Nucleic Acid Ther. 2020 Aug 6;30(4):198–206. doi: 10.1089/nat.2019.0837 (PMC7415887; doi:10.1089/nat.2019.0837)
Supplement: Supplemental data [file Supp_TableS2.pdf]

Supplementary Table S2. Study Treatment Sequence

Treatment sequence	*Period 1*	*Period 2*	*Period 3*	*Period 4*
1: ABPM	300 mg volanesorsen SC +Placebo IV	300 mg volanesorsen IV +Placebo SC	Placebo IV +Placebo SC	400 mg moxifloxacin PO +Placebo IV +Placebo SC
2: BMAP	300 mg volanesorsen IV +Placebo SC	400 mg moxifloxacin PO +Placebo IV +Placebo SC	300 mg volanesorsen SC +Placebo IV	Placebo IV +Placebo SC
3: PAMB	Placebo IV +Placebo SC	300 mg volanesorsen SC +Placebo IV	400 mg moxifloxacin PO +Placebo IV +Placebo SC	300 mg volanesorsen IV +Placebo SC
4: MPBA	400 mg moxifloxacin PO +Placebo IV +Placebo SC	Placebo IV +Placebo SC	300 mg volanesorsen IV +Placebo SC	300 mg volanesorsen SC +Placebo IV
5: BPAM	300 mg volanesorsen IV +Placebo SC	Placebo IV +Placebo SC	300 mg volanesorsen SC +Placebo IV	400 mg moxifloxacin PO +Placebo IV +Placebo SC
6: PMBA	Placebo IV +Placebo SC	400 mg moxifloxacin PO +Placebo IV +Placebo SC	300 mg volanesorsen IV +Placebo SC	300 mg volanesorsen SC +Placebo IV
7: ABMP	300 mg volanesorsen SC +Placebo IV	300 mg volanesorsen IV +Placebo SC	400 mg moxifloxacin PO +Placebo IV +Placebo SC	Placebo IV +Placebo SC
8: MAPB	400 mg moxifloxacin PO +Placebo IV +Placebo SC	300 mg volanesorsen SC +Placebo IV	Placebo IV +Placebo SC	300 mg volanesorsen IV +Placebo SC
9: PABM	Placebo IV +Placebo SC	300 mg volanesorsen SC +Placebo IV	300 mg volanesorsen IV +Placebo SC	400 mg moxifloxacin PO +Placebo IV +Placebo SC
10: AMPB	300 mg volanesorsen SC +Placebo IV	400 mg moxifloxacin PO +Placebo IV +Placebo SC	Placebo IV +Placebo SC	300 mg volanesorsen IV +Placebo SC
11: BPMA	300 mg volanesorsen IV +Placebo SC	Placebo IV +Placebo SC	400 mg moxifloxacin PO +Placebo IV +Placebo SC	300 mg volanesorsen SC +Placebo IV
12: MBAP	400 mg moxifloxacin PO +Placebo IV +Placebo SC	300 mg volanesorsen IV +Placebo SC	300 mg volanesorsen SC +Placebo IV	Placebo IV +Placebo SC

A, volanesorsen 300 mg SC; B, volanesorsen 300 mg IV; M, moxifloxacin 400 mg PO; P, placebo; PO, oral (per os).
