# Supplementary figures and images for: Treatment with Volanesorsen, a 2′-O-Methoxyethyl-Modified Antisense Oligonucleotide Targeting APOC3 mRNA, Does Not Affect the QTc Interval in Healthy Volunteers
Source: Nucleic Acid Ther. 2020 Aug 6;30(4):198–206. doi: 10.1089/nat.2019.0837 (PMC7415887; doi:10.1089/nat.2019.0837)

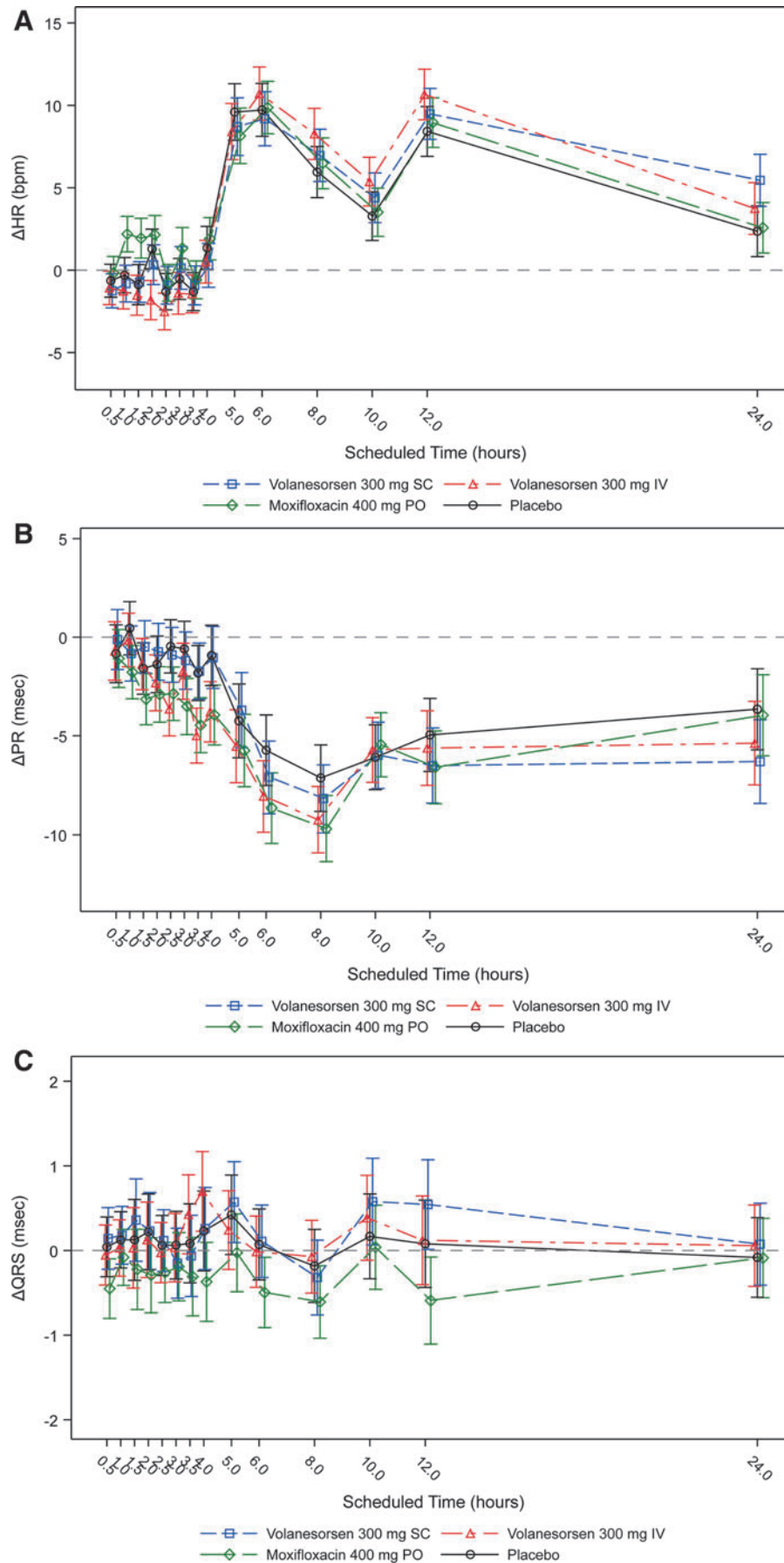

**SUPPLEMENTARY FIG. S1.** Lack of effects on (A) HR, (B) PR or (C) QRS in the volanesorsen QTc study.

Supplement: Supplemental data [file Supp_FigS1.pdf]
