# Supplementary material for: Treatment with Volanesorsen, a 2′-O-Methoxyethyl-Modified Antisense Oligonucleotide Targeting APOC3 mRNA, Does Not Affect the QTc Interval in Healthy Volunteers
Source: Nucleic Acid Ther. 2020 Aug 6;30(4):198–206. doi: 10.1089/nat.2019.0837 (PMC7415887; doi:10.1089/nat.2019.0837)
Supplement: Supplemental data [file Supp_TableS3.pdf]

Supplementary Table S3. Exposure/Response Analysis of Volanesorsen and Associated ΔΔQTcF Prolongation (PK/QTc Population)

Model	*AIC*	*Parameter*	*Estimate (90% CI)*	P	*Between-subject variation*
1	6420	Intercept (ms)	0.742121 (–0.527952 to 2.012195)	0.3323	4.640383
		Slope (ms per ng/mL)	–0.000024 (–0.000054 to 0.000006)	0.1827	
		Residual variability (ms)	7.1396197		
2	6419	Intercept (ms)	0 fixed	0.2746	4.680000
		Slope (ms per ng/mL)	–0.000019 (–0.000048 to 0.000010)		
		Residual variability (ms)	7.140000		
3	6540	Slope (ms per ng/mL)	–0.0000002 (–0.000051 to 0.000047)	0.9459	0.000000
		Residual variability (ms)	7.790000		

AIC, Akaike information criterion; CI, confidence interval.
